# Tuning Coherent-Phonon Heat Transport in LaCoO_3_/SrTiO_3_ Superlattices

**DOI:** 10.1021/acs.jpclett.1c03418

**Published:** 2021-12-07

**Authors:** D. Bugallo, E. Langenberg, E. Carbó-Argibay, Noa Varela Dominguez, A. O. Fumega, V. Pardo, Irene Lucas, Luis Morellón, F. Rivadulla

**Affiliations:** † Centro Singular de Investigación en Química Biolóxica e Materiais Moleculares (CIQUS), Departamento de Química-Física, Universidade de Santiago de Compostela, 15782 Santiago de Compostela, Spain; ‡ Department of Condensed Matter Physics, Institute of Nanoscience and Nanotechnology (IN2UB), University of Barcelona, 08020 Barcelona, Spain; § International Iberian Nanotechnology Laboratory (INL), Av. Mestre José Veiga s/n, 4715-330 Braga, Portugal; ∥ Departamento de Física Aplicada, Universidade de Santiago de Compostela, 15782 Santiago de Compostela, Spain; ⊥ Department of Applied Physics, Aalto University, FI-00076 Aalto, Finland; ∇ Instituto de Nanociencia y Materiales de Aragón (INMA), Universidad de Zaragoza and Consejo Superior de Investigaciones Científicas, 50009 Zaragoza, Spain

## Abstract

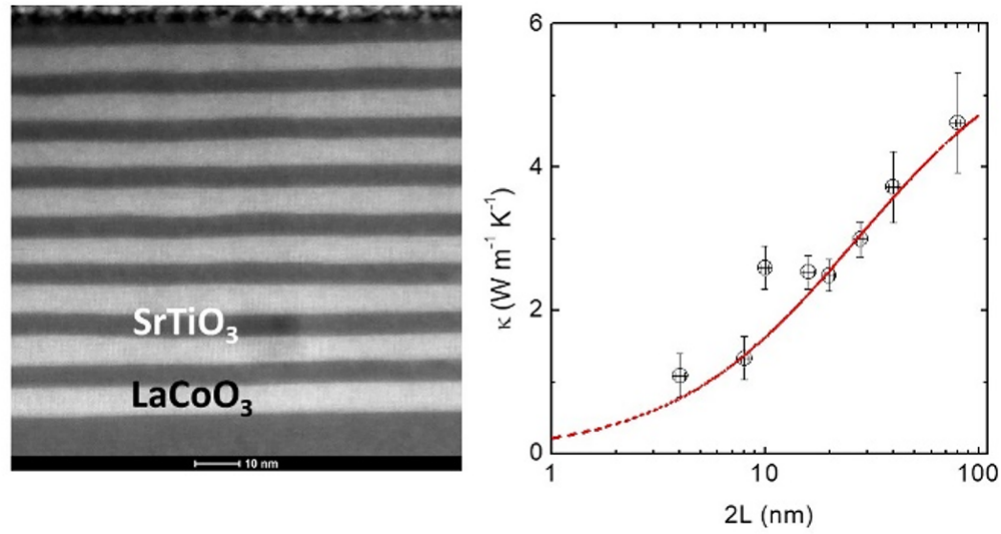

Accessing the regime of coherent phonon propagation in nanostructures opens enormous possibilities to control the thermal conductivity in energy harvesting devices, phononic circuits, etc. In this paper we show that coherent phonons contribute substantially to the thermal conductivity of LaCoO_3_/SrTiO_3_ oxide superlattices, up to room temperature. We show that their contribution can be tuned through small variations of the superlattice periodicity, without changing the total superlattice thickness. Using this strategy, we tuned the thermal conductivity by 20% at room temperature. We also discuss the role of interface mixing and epitaxial relaxation as an extrinsic, material dependent key parameter for understanding the thermal conductivity of oxide superlattices.

There are three important length scales whose relative size determines the lattice thermal conductivity, κ, in nanostructures: the phonon mean free path, *l*, their wavelength, λ, and a characteristic physical length of the system, *L* (the period length in a multilayer, for instance).^1^ For periodically arranged interfaces, as in a superlattice (SL), phonons of sufficiently long-wavelength (λ > *L*) may undergo wave-interference effects, given their *l* is long enough to propagate over several interfaces (i.e., *l* > *L*). In this regime, phonons behave as coherent waves, with a ω(*k*) dispersion characteristic of the SL, with their group velocity and density of states for each polarization, as well as energy gaps that forbid the propagation of certain phonon frequencies, decreasing κ of the SL as L increases.^2^ Coherent propagation of thermal phonons was demonstrated by Luckyanova et al.^3^ in GaAs/AlAs SLs, taking advantage of the long λ and *l* in these semiconductors. Control of κ through wave-interference effects has been also achieved in Si nanostructures with periodically arranged patterns, spaced ≈*l*.^4−6^ Note that the high sensitivity of wave-interference effects to the periodicity of the SL, introduces another tunable parameter to control the thermal conductivity of a nanostructure, at a length scale that should not affect much the electrical conductivity, raising the interest for thermoelectric applications.^7^

On the other hand, a progressive increase of *L* will put more phonons at *l* < *L*, increasing the contribution from incoherent phonons to κ.^8−12^ Thus, the crossover from a regime in which heat transport is governed by coherent phonons, to another one in which incoherent phonons dominate, should, in principle, be signaled by a minimum in the thermal conductivity of the SL at a given *L*.^2,13^

Most of the experimental work to corroborate this crossover has been carried out in semiconductor SLs, due to their large mean free path, the possibility of growing clean interfaces (defects of the order of λ produce diffuse reflections and loss of phonon-phase coherence, resulting in particle-like, incoherent, propagation), and their interest in thermoelectric applications.

For instance, a minimum at κ(*L*) was reported by Chakraborty et al.^14^ in Si–Ge SLs at *L* ≈ 7 nm; Venkatasubramanian^15^ also observed the crossover in Bi_2_Te_3_/Sb_2_Te_3_ superlattices, at *L* ≈ 5 nm. However, other authors did not find evidence of the minimum at κ(*L*) in Bi_2_Te_3_/Sb_2_Te_3_ or GaAs/AlAs SLs, suggesting a critical effect of interface roughness.^16−19^ Comparing the results from GaAs/AlAs SLs of different thickness and periods, Cheaito et al.^18^ confirmed the contribution of both incoherent and coherent phonons, even in the absence of the minimum κ(*L*); similar conclusions were reached by Luckyanova et al.^20^ and Alaie et al.,^6^ the latter in Si membranes.

Regarding oxide multilayers, a shallow minimum at *L* = 2–3 nm was reported by Ravichandran et al.^21^ in SLs of SrTiO_3_/CaTiO_3_ and SrTiO_3_/BaTiO_3_. On the other hand, Katsufuyi et al. observed a linear increase of κ(*L*) in SrTiO_3_/SrVO_3_ SLs, from *L* ≈ 4 to 100 nm,^22^ without any sign of κ(*L*) minimum or flattening at low *L*. Instead, these authors reported a constant interfacial resistance ≈2 × 10^–9^ K m^2^/W. A monotonic decrease was also observed in the thermal conductivity of (SrTiO_3_)_*n*_SrO Ruddlesden–Popper superlattices, as the interface density increases.^23^

Transition-metal 3d oxides are quite ionic, and sharp interfaces may introduce polar discontinuities in some cases, whose energy penalty can be resolved through ionic intermixing.^24^ This, together with their shorter *l* and λ than semiconductors, should make them more sensitive to interfacial defects.^25,26^ Thus, the observation of a minimum κ(*L*) in oxide SLs should be more difficult than in semiconductors and places the question of how relevant wave-interference effects are in oxide SLs and how much their thermal conductivity may be tuned acting over coherent-phonons.

Here we report a systematic study of LaCoO_3_/SrTiO_3_ (LCO/STO) SLs, varying the total thickness *t*, and the lattice period, *L*, as well as the periodicity of the structures. We show that both coherent and incoherent phonons contribute to κ of the SL, at all periodicities. We also demonstrate that the contribution of long-wavelength coherent phonons can be reduced by the effect of small variations of the periodicity, even at large *L* and at room temperature, which could be useful in thermoelectrics and in thermal management devices.

A series of LaCoO_3_/SrTiO_3_ (LCO/STO) SLs with a total thickness of *t* = 80 nm and different periods were synthesized by PLD (see Table S1, Supporting Information). We denoted our SLs by (*L* × *n*), where *L* is the thickness of each individual layer forming the SL (so that the period of the superlattice is 2*L*) and *n* is the repetition of each layer. The materials for the SL of this study, STO, *a*_STO_ = 3.905 Å, and LCO, *a*_LCO_ = 3.80 Å, were selected, under the premise of having different enough masses and lattice constants, but still showed a good epitaxial growth on top of each other, to have good crystallinity and well-defined interfaces. The cumulative thermal conductivity calculated ab initio, Figure S1 in the Supporting Information, showed that phonons have similar mean free paths in STO and LCO. Also, from these data, a substantial effect is expected for periods 2*L* ≈ 10–20 nm, between 100 and 250 K. Thus, LaCoO_3_ and SrTiO_3_ seem a good compromise for studying the contribution of coherent/incoherent phonon transport in epitaxial oxide SLs.

The structural and microstructural analysis of the samples is summarized in Figure 1. The Θ–2Θ X-ray diffraction patterns around the (002) peak of the STO substrate show an increasing number of periodically spaced SL peaks as the number of periods increases, indicating the preservation of the long-range order. The satellite peaks along the Q_*z*_ axis in the reciprocal space maps (RSM, Figure 1b) further confirm the periodic structure along the out-of-plane direction of the SL and show that the SLs are coherently strained with the substrate—see also the discussion of the scanning transmission electron microscopy (STEM) data below. X-ray reflectivities show the characteristic SL peaks, as well as a smooth angular decay, suggesting smooth interfaces. The thickness of the SLs obtained from these analyses are in good agreement with each other and correlate very well with the nominal thickness (see Table S1 in the Supporting Information, and Figure S2, for further details of the fittings and results of the structural analysis).

**Figure 1:**
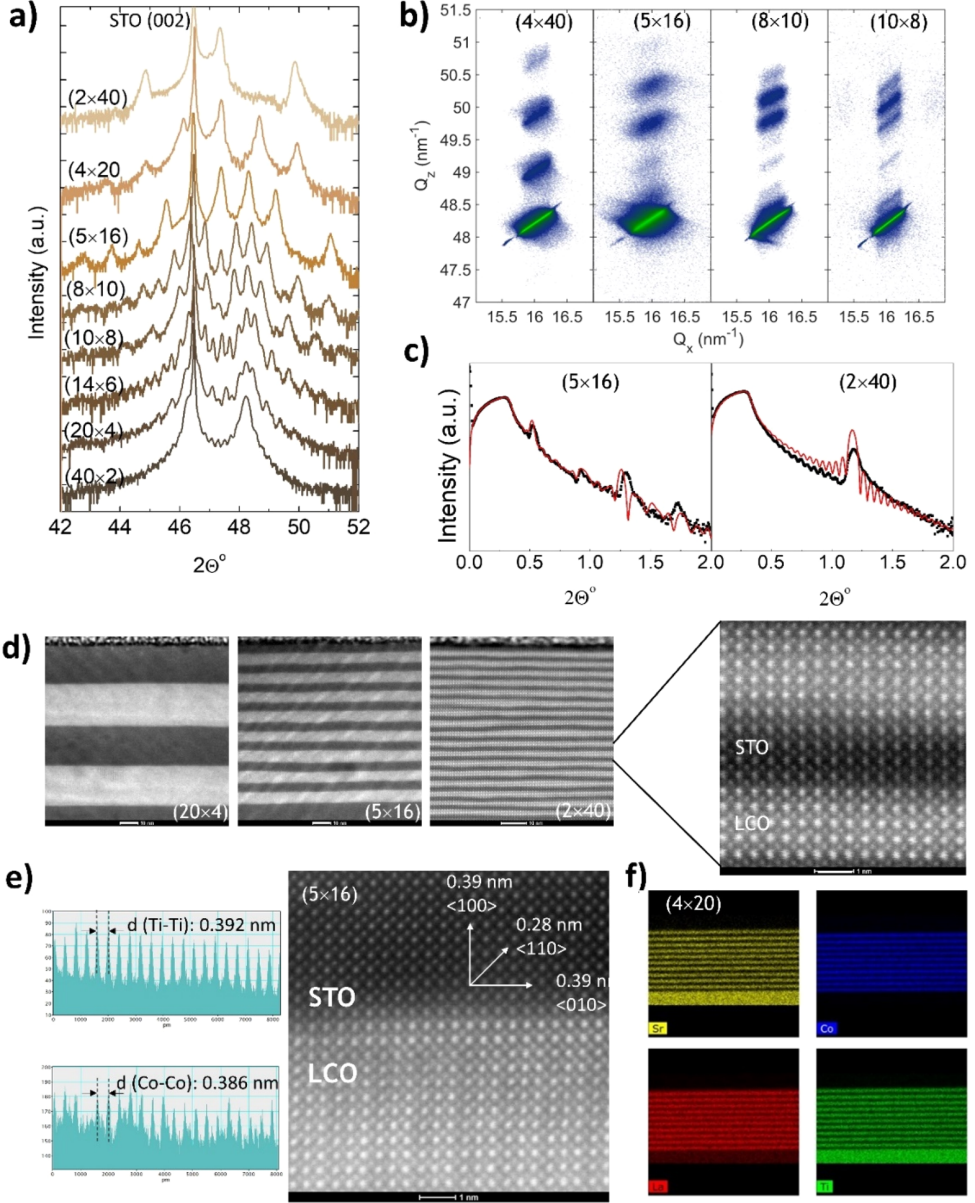
(a) Θ–2Θ X-ray diffraction pattern of the SLs around the (002) peak of the (001)-oriented STO substrate. The total thickness of each SL is *t* ≈ 80 nm. The periodicity is indicated in each case. (b) RSM around the (103) reflection of the STO substrate. The periodicity is indicated in each panel. (c) X-ray reflectivity of two samples showing the SL peaks and smaller oscillations related to the total thickness. (d) Cross section high-angle annular dark-field (HAADF)-STEM images of several SLs, with the period indicated on each panel. The scale (white bar) is 10 nm in every image, except in the zoomed area of the (2 × 40) SL, right, which is 1 nm. (e) Image intensity profiles (displaying Sr–Sr and Co–Co spacings) parallel to the plane of the sample, and HAADF-STEM image from a cross-section lamella of a (5 × 16) SL, showing the crystalline structure. The metal–metal distance obtained from the image intensity profiles analysis is ≈3.92 Å for Ti–Ti (STO) and ≈3.86 Å for Co–Co (LCO), denoting a slight relaxation in STO. (f) EDX map analysis of a lamella from a (4 × 20) SL, showing the regularity of the layer thickness. The total thickness of the SL is ≈79 nm, giving an average of 3.95 nm per layer, very close to the intended 4 nm per layer.

The microstructure of the internal interfaces was studied by high resolution STEM on several cross-section lamellae of different SLs. The results (Figure 1d–f), show that LCO and STO grow epitaxially on top of each other, with very-well-defined interfaces, and with a thickness very close to the nominal ones (see also Figures S2 and S3 in the Supporting Information). The width of the interfaces, defined as the region where the intensity of the EDX peaks (Ti and Co) decays at half its maximum value, is of the order of one-two unit cells (Supporting Information Figure S3). Therefore, from the X-ray diffraction and STEM analysis, we conclude that the LCO/STO SLs present an excellent crystallinity and clean interfaces, free from a substantial number of defects that could affect our analysis of their intrinsic thermal conductivity.

The cross-plane κ(*T*) of the SLs was measured from 25 to 290 K by the 3ω method^27^ (see Supporting Information for details of the measurements); the results are shown in Figure 2 for the SLs with total thickness *t* = 80 nm. In all SLs, κ(*T*) first increases up to 150–200 K, before reaching a plateau, and then decreases slightly until room temperature in the SLs with thicker periods.

**Figure 2:**
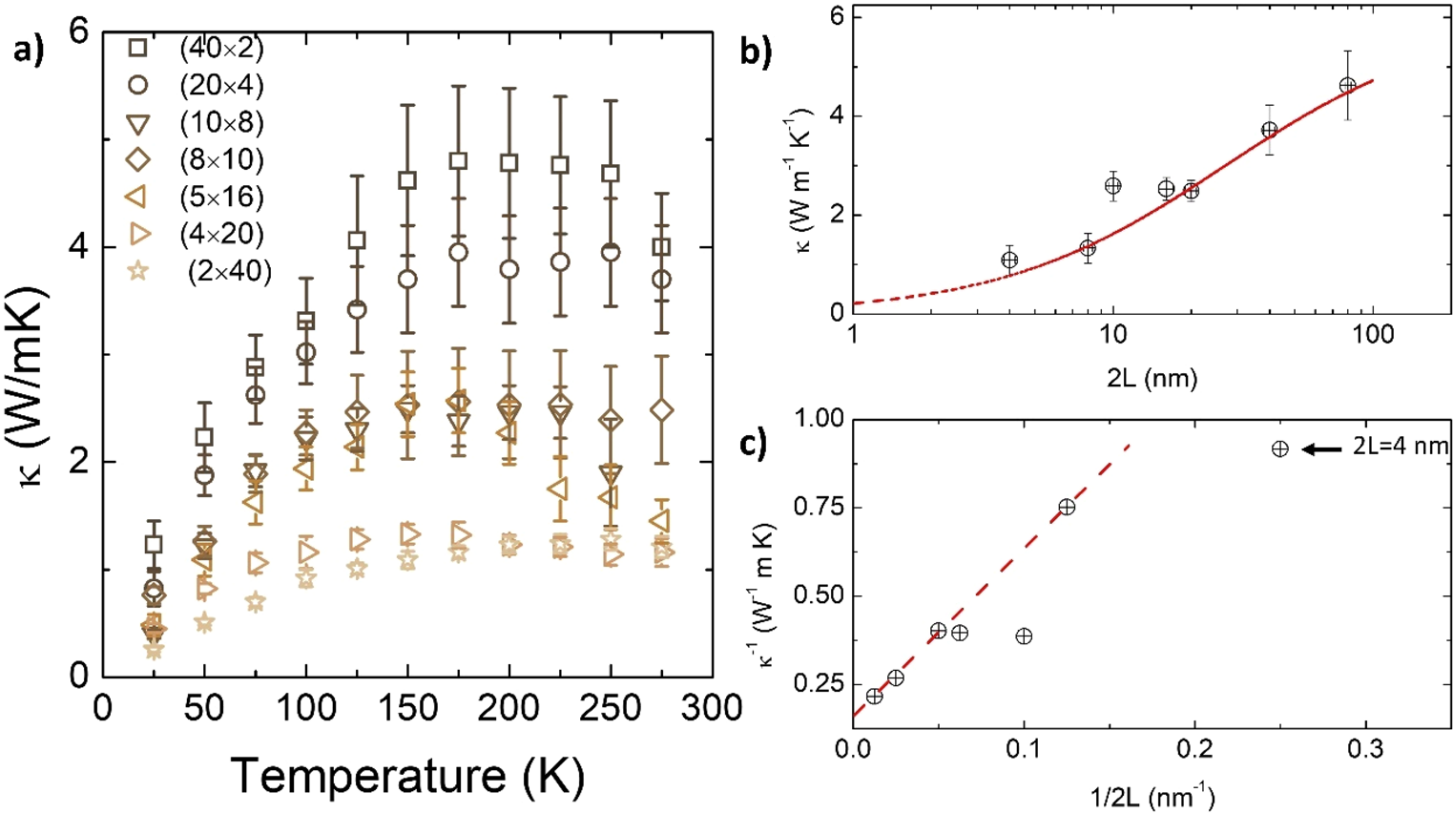
(a) Temperature dependence of the thermal conductivity of the different SLs with *t* = 80 nm (see Table S1 in the Supporting Information for further details of the periodicity of each sample). (b) Dependence of the thermal conductivity of the SLs at 150 K with the period 2*L* and the fitting to eq 1. (c) Linearized version of eq 1 and linear fitting of the experimental data. As discussed in the text, the validity of eq 1 at low values of *L* is compromised, when the Kaptiza and the period lengths, become comparable. For that reason, the data at 2*L* = 4 (marked with an arrow), was excluded from the fitting.

In Figure 2b,c we present the cross-plane κ at 150 K vs the SL period, 2*L*: the thermal conductivity of the SL decreases as 2*L* does, indicating that *l* must be comparable to the SL period. Therefore, at least for a significant portion of phonons, the effect of the interface boundary resistance can be captured by a simple model incorporating the interfacial Kapitza resistance, *R*_*if*_, into the Fourier’s law of heat conduction across the SL:^8,9^
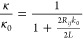
1κ_0_ and *R*_*if*_ represent the thermal conductivity of the bulk, free of interfaces, and the interfacial thermal resistance, respectively. This equation predicts a linear relationship between 1/κ and 1/2*L*; however, as shown in Figure 2b, the data for 2*L* below ≈20 nm deviate progressively from this behavior. Fitting the data for 2*L* > 10 to eq 1 gives κ_0_ ≈ 6.25(8) W m^–1^ K^–1^, close to the average of STO and LCO at this temperature (see Figures S4–S6 in the Supporting Information for the thermal conductivity of individual LCO and STO thin films, as well as for a short discussion of the accuracy of the thermal conductivity measurements), and *R*_*if*_ ≈ 4.7(2) × 10^–9^ W^–1^ m^2^ K, similar to other oxoperovskite artificial interfaces, grain boundaries, or ferroelastic domain walls.^22,28,29^ Note, however, that the Kapitza length, *L*_*K*_ = *R*_*if*_*k*_0_ ≈ 24 nm, becomes comparable to, or even larger than, the period length for SLs at 2*L* < 20 nm; below this limit, the applicability of eq 1 is not justified. Instead, the reduction of the period thickness makes *l* > 2*L* for an increasing population of phonons, so they become less sensitive to the periodicity of the internal interfaces of the SL. In this case, a wave-like treatment is probably more appropriate and κ is determined by wave interference and boundary scattering at the external interfaces of the SL. In fact, below 2*L* < 20 nm, there is a departure from the prediction of eq 1, manifested as a plateau around 2*L* = 10–20 nm, and a larger than expected κ for 2*L* = 4 nm (better appreciated in the linearized plot of Figure 2c). In any case, an actual minimum in κ(2*L*) is not observed.

As discussed before, atomic intermixing at the interfaces of thinner period SLs could be large enough to produce diffusive scattering. Although electron microscopy showed sharp interfaces, roughness is a statistical quantity, and STEM only probes a very limited region (nanometer size) of a much wider sample (mm size). Therefore, to further asses the quality of the interfaces we measured the bulk magnetic properties of the SLs.

Tensile strained LCO develops a magnetic order below *T*_C_ ≈ 85 K and a coercive field at low temperatures up to ≈10 kOe.^30,31^ The SLs with thicker layers of LCO, i.e., *L* ≥ 10 nm (2*L* > 20 nm), show similar behavior to a 40 nm thin film of LCO on STO, with a slight decrease of *T*_C_ and saturation magnetization (Figure 3a,b). However, the magnetic behavior changes at *L* ≤ 10 nm, with a strong reduction of the saturation magnetization and coercive field (Figure 3c), and the appearance of a (probably antiferrro) magnetic signal around ≈68 K.

**Figure 3:**
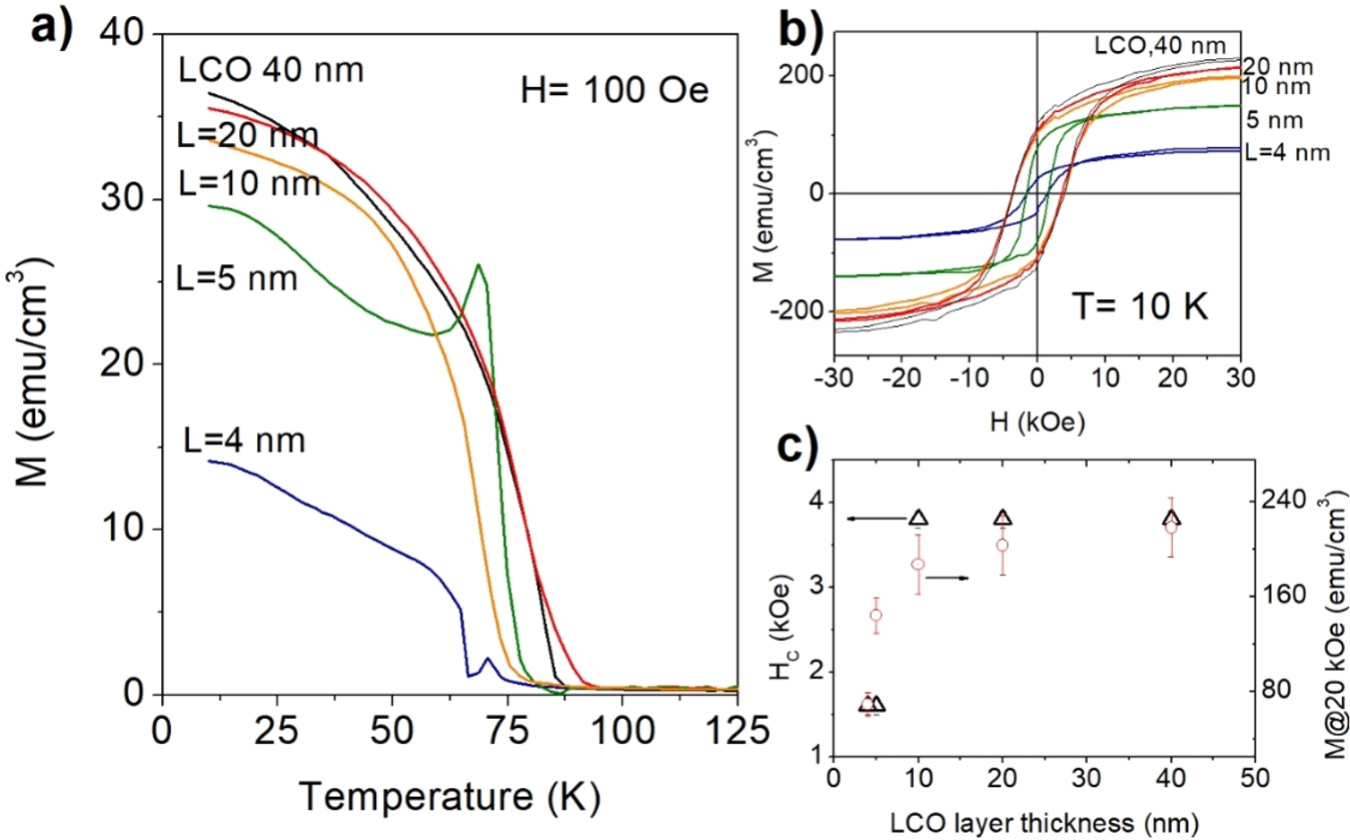
Temperature dependence of the magnetization measured at a magnetic field *H* = 100 Oe (a), and hysteresis loops at 10 K (b) of several LCO/STO SLs. A thin film of LCO (40 nm) on STO is also shown for comparison. (c) Coercive field (triangles), *H*_C_, and magnetization at 20 kOe (cricles), both obtained from the hysteresis loops in (b). There is a rapid decrease of both magnitudes in the films with *L* < 10 nm.

Zhang et al.^32^ reported a change in the oxygen vacancy pattern for LCO on STO thin films thicker than 5 nm, signaling a change in the mechanisms of relaxation of epitaxial stress at this critical thickness. Also, Zhang et al.^33^ found a suppression of the characteristic ferromagnetic phase of strained LCO in LCO/STO SLs with LCO layers thinner than ≈6 nm.

Thus, our results, particularly the magnetic signal at 68 K, point toward the existence of an additional interlayer region for *L* ≤ 10 nm, whose composition cannot be determined from our data but could be a mixed phase of the type (Sr,La)(Co,Ti)O_*x*_. Several magnetic oxides of Sr–Co and Ti–Co are reported in the literature, like Co_2_TiO_4_ and CoTiO_3_, with a smaller *T*_C_ than LCO.^34^

However, Ju et al.^35^ showed that mixed interfaces could promote phonon transmission through a more gradual relaxation of epitaxial strain and acoustic mismatch; other types of defects, like oxygen vacancies or disordered cation substitution, could be however more detrimental for κ, as it seems to be the case here.

For an intuitive understanding of the effect of interfacial roughness, η, we defined a dimensionless parameter, *x*, which determines the fraction of coherent/incoherent phonons in the SL: . Note that *x* decreases as *L* and η increase, so that the effective thermal conductivity as a function of the periodicity, 2*L*, is obtained from the weighted fraction of coherent (ballistic) phonons and incoherent phonons following
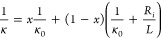
2Although this is a very crude approximation (κ diverges at *L* → 0), it gives an idea of the relevant parameters contributing to κ(*L*) of a SL. The κ(*L*) calculated for different values of the roughness is shown in Figure 4.

**Figure 4:**
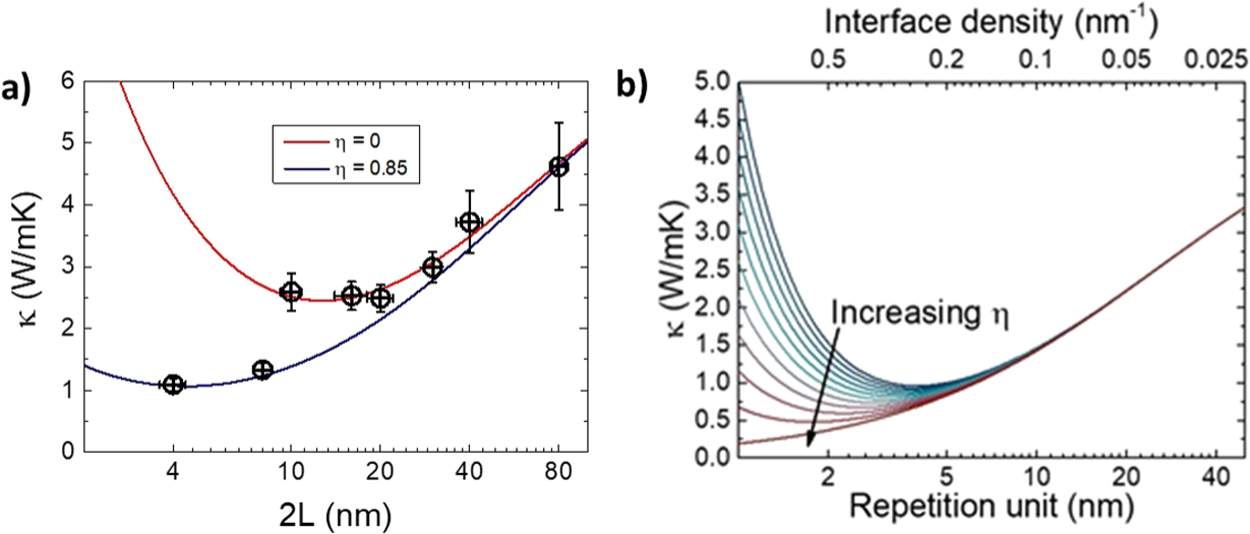
(a) Fitting of experimental κ(150 K) to eq 2, for different values of roughness. (b) Calculation of the thermal conductivity according to eq 2, as a function of the interfacial roughness, and κ_0_ = 4.5 W m^–1^K^–1^.

Equation 2 fits the experimental κ(150 K, *L*) data of the SLs down to 2*L* ≈ 10 nm with a roughness η ≈ 0 and *R*_*i*_ = 3.57 × 10^–9^ K m^2^/W. The fitting also suggests that the plateau (or local minimum) of κ(*L*) around this region could be consistent with a vanishing interfacial rugosity, which increases due to ionic interdiffusion as *L* decreases, according to magnetic data.

An increasing η does not affect κ(*L*) at large *L*, where incoherent phonon transport is dominant, and the contribution of coherent phonons at low *L* becomes more relevant as η decreases. The equation shows the existence of a minimum at the crossover between those regimes, if η is small enough.

To probe the contribution of coherent phonons below/above the 2*L* ≈ 10 nm boundary, we prepared additional sets of SLs with different total thicknesses but keeping their periodicities. In Figure 5b we compare the thermal conductivity of a SL with 2*L* = 40 nm (2*L* > *L*_*K*_) and another one with 2*L* = 8 nm (2*L < L*_*K*_), varying the total thickness, *t*.

**Figure 5:**
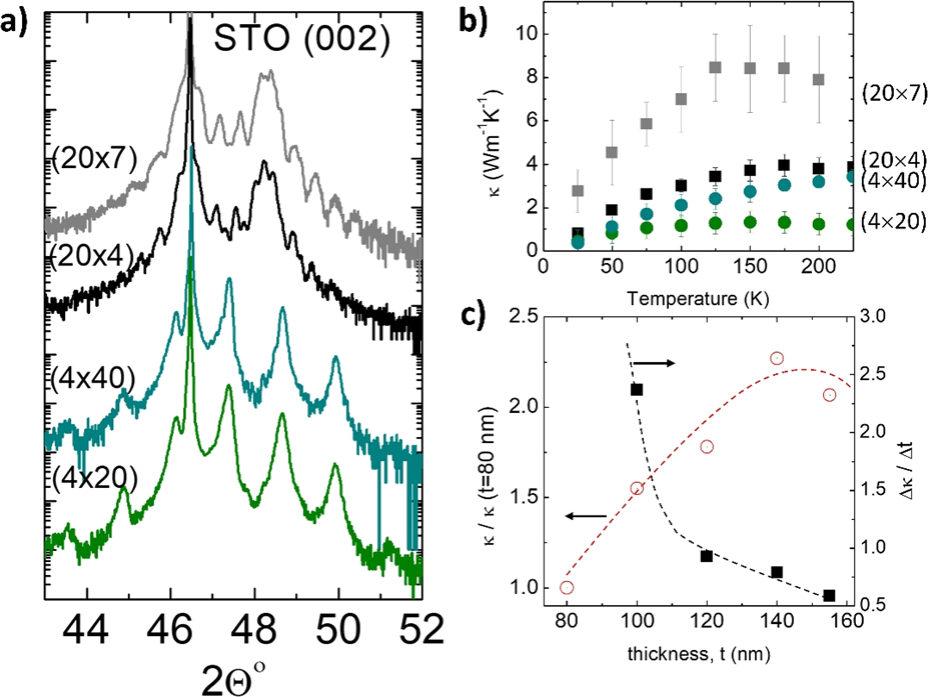
(a) XRD of the SLs with *L* = 4 nm and *L* = 20 nm, with different total thicknesses. The period of the SL is maintained, as shown by the similar superlattice peaks in the XRD pattern. (b) Temperature dependence of the thermal conductivity for the two sets of SLs. (c) Thermal conductivity at 150 K for the SLs, with different total thickness (open symbols), and relative increase of the thermal conductivity with respect to the 80 nm thick films, normalized by the relative increase of thickness: . Lines are guides to the eye.

The results show that κ increases with *t*, at a similar rate, suggesting that, irrespective of the SL periodicity, there is a portion of phonons whose mean free path is limited by the total thickness of the SL and must be treated as coherent waves. On the other hand, the relative increase of κ, normalized by the thickness of the SL, decreases continuously (Figure 5c) and becomes less than *l* at *t* ≈ 120 nm. Beyond that point, increasing the SL thickness is not compensated by the contribution of larger mean free path phonons, and the probability of anharmonic phonon–phonon scattering increases sufficiently to reduce their contribution to the thermal conductivity. These results show that coherent phonons with a maximum *l* ≈ 120 nm contribute substantially to the thermal conductivity of LCO/STO SLs.

This opens the possibility to reduce the thermal conductivity of oxide SLs at large 2*L*, without introducing a large density of (or rough) interfaces, which is an interesting strategy for oxide-based thermoelectric devices. To probe this hypothesis, we prepared a SL with an average *L* = 14 nm, but with an intentional aperiodicity of 15–20% between neighboring layers, (14 × 6):ap; see Figure 6. Long wavelength coherent phonons should be very much affected by a change in the periodicity of the SL,^35,36^ while particle-like phonons should remain insensitive to it, as long as 2*L* > *l*.

**Figure 6:**
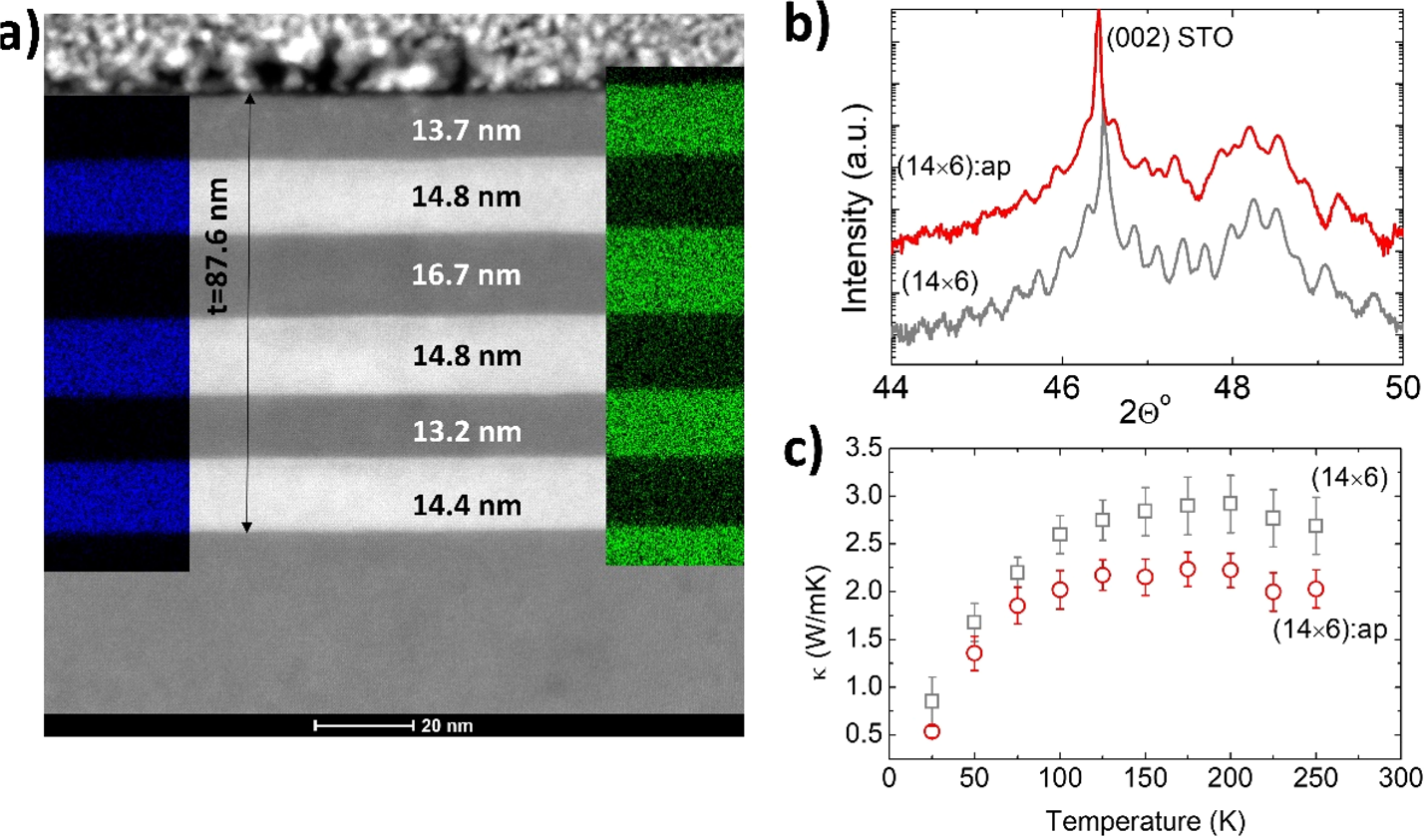
(a) HAADF-STEM and EDX analysis of cross section lamellae of the aperiodic SL, (14 × 6):ap. The blue/green signal of the EDX map corresponds to Co/Ti, respectively. The X-ray diffraction pattern of the aperiodic and regular (14 × 6) SLs, is shown in (b). The loss of periodicity is clearly reflected in the disappearance of many of the SL peaks on the (14 × 6):ap. (c) Temperature dependence of the thermal conductivity of both SLs.

Despite the high quality of the interfaces, the X-ray diffraction pattern in Figure 6b shows the partial loss of the SL peaks in the aperiodic structure. The interferences between X-ray beams that produce SL peaks in the X-ray diffraction pattern have similar origins as the phonon wave interference, although a broad range of phonon frequencies contribute to κ. Similarly to that loss of periodicity, the thermal conductivity of the SL:ap is reduced by ≈25%, between 100 and 250 K. This result shows that even small variations in the periodicity may be a valid approach to control the thermal conductivity of SLs through the suppression of coherent phonons, even for a relatively small number of repetitions.

In summary, we have shown that the contribution of coherent phonons to oxide SLs is relevant in the whole period length and can be substantially reduced by small variations of the periodicity, without affecting its total thickness. Using this strategy, we tuned the thermal conductivity of LaCoO_3_/SrTiO_3_ SLs up to 20% at room temperature. This may have an interesting application in the development of low thermal conductivity devices, in which maintaining a relatively large thickness and clean interfaces is important for not deteriorating electrical transport, as in thermoelectrics.

## Supporting Information Available

List of samples studied in this work, including their total thickness and periodicity (Table S1), all the details about the deposition procedure, cumulative thermal conductivity of SrTiO_3_ and LaCoO_3_, calculated ab initio (Figure S1), details of the structural and compositional analysis of the samples, through X-ray diffraction and electron microscopy (Figures S2 and S3), complete discussion about the accuracy of the thermal conductivity measurements by the 3ω method (Figures S4 and S5), with the temperature dependence of the thermal conductivity of the SrTiO_3_ and LaCoO_3_ thin films (Figure S6) (PDF)

## Notes

All data and materials used in the analyses are available under reasonable request to the authors.
