# Supplementary material for: Tuning Coherent-Phonon Heat Transport in LaCoO3/SrTiO3 Superlattices
Source: J Phys Chem Lett. 2021 Dec 7;12(49):11878–85. doi: 10.1021/acs.jpclett.1c03418 (PMC8686111; doi:10.1021/acs.jpclett.1c03418)
Supplement: Supplementary file 1 — jz1c03418_si_001.pdf [file jz1c03418_si_001.pdf]

Supporting Information for:

Tuning Coherent-Phonon Heat Transport in $LaCoO_3/SrTiO_3$ Superlattices.

*D. Bugallo,[1] E. Langenberg,[2] E. Carbó-Argibay,[3] Noa Varela Dominguez,[1] A. O. Fumega,[4,5] V. Pardo,[4] Irene Lucas,[6] Luis Morellón,[6] F. Rivadulla.[1,]**

[1]Centro de Investigación en Química Biolóxica e Materiais Moleculares (CIQUS) and Departamento de Química-Física, Universidade de Santiago de Compostela, 15782 Santiago de Compostela, Spain.

[2]Department of Condensed Matter Physics, Institute of Nanoscience and Nanotechnology (IN2UB), University of Barcelona, 08020 Barcelona, Spain.

[3]International Iberian Nanotechnology Laboratory (INL), Av. Mestre José Veiga s/n, 4715-330 Braga, Portugal.

[4]Departamento de Física Aplicada, Universidade de Santiago de Compostela, 15782 Santiago de Compostela, Spain.

[5]Department of Applied Physics, Aalto University, FI-00076 Aalto, Finland

[6]Instituto de Nanociencia y Materiales de Aragón (INMA), Universidad de Zaragoza and Consejo Superior de Investigaciones Científicas, 50009 Zaragoza, Spain.

**e-mail:* f.rivadulla@usc.es

Pulser Laser Deposition of the superlattices.

The $LaCoO_3/SrTiO_3$ (LCO/STO) SLs were deposited on (001)-oriented $SrTiO_3$ single crystals, used as received. The first layer of the SLs was always LCO, and the top layer was always STO. The thickness of each layer, and the total thickness of the SLs studied in this work, are reported in Table S1.

The laser for the PLD growth is a frequency-quadrupled Nd:YAG laser with a wavelength of 266 nm and maximum energy of 100 mJ. The fluence of the laser to grow the LCO and STO films was ≈ 2.3 J/cm^2. The substrate was kept at 725°C during deposition, and in an oxygen pressure of 200 mTorr. After deposition, the samples were cooled at 5°C/min in the same atmosphere. The growth rate (calibrated via ex-situ X-ray reflectivity analysis, for each material) with these conditions was 0.024 Å/pulse for STO and 0.027 Å/pulse for LCO.

Table S1. List of samples grown for this work. The nominal thickness of each layer, L, and total thickness, t, of the SLs synthesized for this work, as well as the experimental values obtained from different experimental methods.

**Sample**	**Layer thickness L (nm)**	**Total SL thickness t (nm)**	**Thickness XRD (nm)**	**Thickness XRR (nm)**	**Rugosity XRR (nm)**
**(2×40)**	2	80	1.9	1.96	0.5(1)
**(4×20)**	4	80	3.8	3.8	0.8(2)
**(4×40)**	4	160	3.8	3.9	0.5(1)
**(5×16)**	5	80	5.4	5.3	0.7(3)
**(5×24)**	5	120	5.7	5.6	0.9(3)
**(8×10)**	8	80	9.0	7.8	1.1(3)
**(10×8)**	10	80	10.5	10.4	0.7(2)
**(14×6)**	14	84	17.5	15.2	1.4(2)
**(14×6):ap**	≈13-16	≈ 88 (TEM)			
**(20×4)**	20	80	20.9	21.5	1.3(2)
**(20×6)**	20	120	19.6	19.5	1.5(3)
**(40×2)**	40	80	40.2	46.1	0.9(1)

Ab-initio calculation of the cumulative thermal conductivity.

The ab-initio cumulative thermal conductivity is shown in Figure S1 for LCO and STO, at two different temperatures, vs the phonon mean free path, l_{MFP}. The STO two-step dependence of the cumulative lattice thermal conductivity is in agreement with previous calculations that highlight that

thermal transport is carried not only by the acoustic modes, but also by the polar ones[1]. At 100 K, about 40% of heat is transported by ℓ<20 nm; this amount increases to ≈70 % at 240 K. Thus, between 100 K and room temperature, a periodic structure with a characteristic length 2L≈10-20 nm is expected to have a strong impact in the propagation of a large portion of phonons.

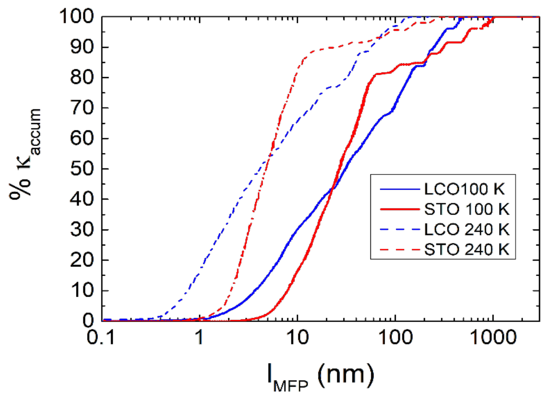

Figure S1. Cumulative thermal conductivity for $SrTiO_3$ and $LaCoO_3$, calculated ab initio.

The first principles density functional theory (DFT) calculations[2,3] were performed for LCO and STO in their low temperature structures. The generalized gradient approximation (GGA) in Perdew-Burke-Ernzerhof (PBE) scheme[4] was used for the exchange-correlation term with the PAW method as implemented in the VASP code[5]. The harmonic interatomic force constants (IFCs) were computed using the density-functional perturbation theory (DFPT)[6]. The third-order anharmonic IFCs were computed using the real-space supercell approach with a 3×3×3 supercell. These IFCs allow to compute the cumulative lattice thermal conductivity by iteratively solving the linearized Boltzmann-Peierls transport equation of phonons with the ShengBTE package[7]. Converged phonon momenta q-meshes of 15×15×15 were used for solving the transport equation of both compounds.

Structural X-ray analysis of the SLs.

X-ray experiments were performed in a PANalytical Empyrean 4 circle diffractometer, equipped with a PIXCel^{3D} detector and a Ge (220) double bounce monochromator, and a Cu source, 1.540598 Å (Cu $K_{\alpha 1}$). In the case of a SL, the X-ray beam reflects on the different interfaces of the multilayer, giving rise to constructive interferences at some angles that are related to the thickness of each layer:

$$2L = \frac{\lambda m}{2(sin(\theta_m) - sin(\theta))} \qquad (S1)$$

where $2L$ is the thickness of the repeating unit in the SL, λ the wavelength, and m is the order of the peak at θ_m, with respect to the center one at θ. The X-ray pattern of the SL formed by 8 repetitions of $SrTiO_3$/$LaCoO_3$, with L=5 nm of thickness in each layer (5×16), is shown in Figure S2, along with the fitting. The period obtained from these analyses is 2L=10.4 nm, close to the nominal one 2L=10 nm. The fit to equation (S1) is shown in Figure S2 also for a (20×4) SL.

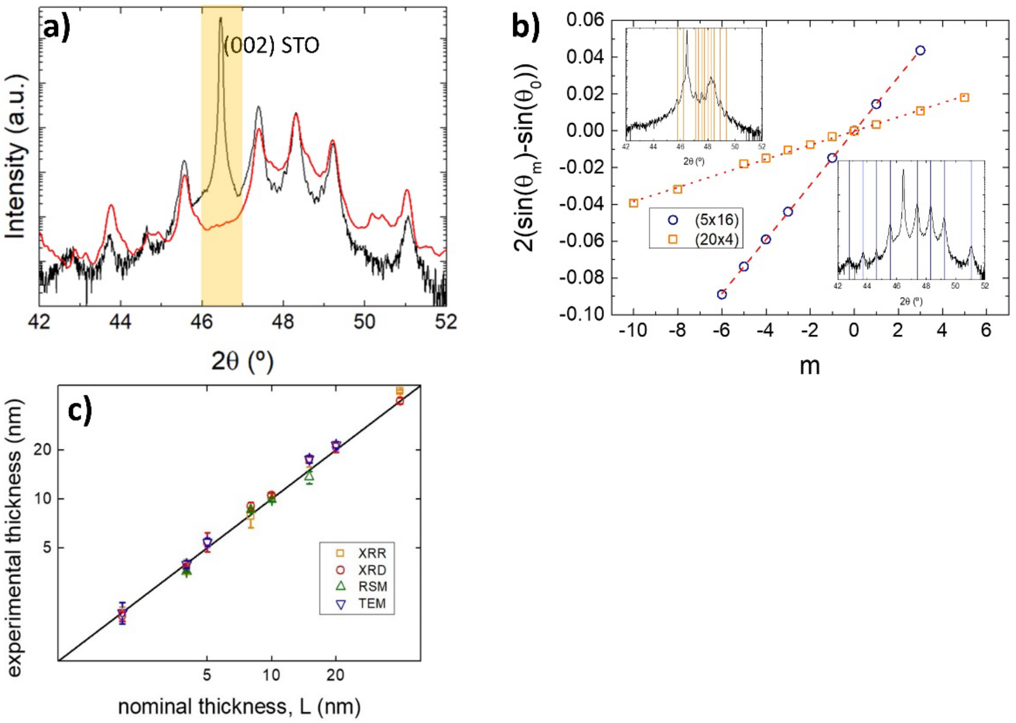

Figure S2. a) X-ray diffraction of a (5×16) SL, and simulation (red line); the peak of the substrate (shaded area) is not simulated. b) X-ray diffraction patterns with the position of the SL peaks marked (insets), and fitting to equation (S1), of the SL (20×4) and (5×16). c) Experimental vs. nominal thickness of each layer (L), obtained by different X-ray analyses, and TEM.

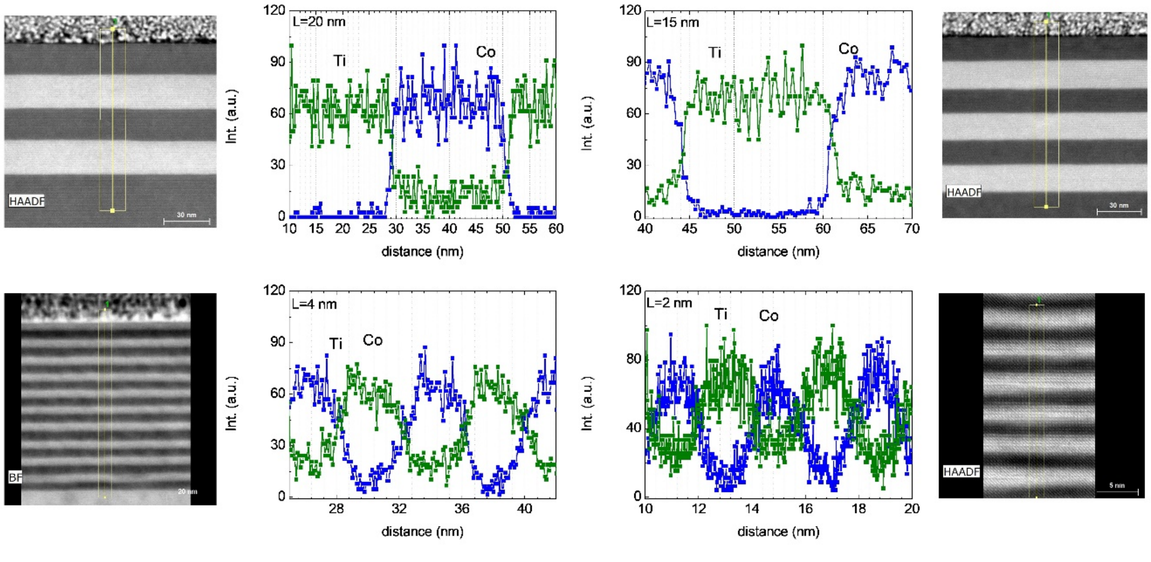

Figure S3. HAADF-STEM and EDX line-scan analysis of several SLs, with (from top, left) L=20 nm, 15 nm, 4 nm, and 2 nm. HAADF-STEM and EDX analysis have been carried out in a probe- and

image-corrected FEI Titan Themis microscope operated at 200 kV. Previously to HAADF-STEM and EDX analysis, a cross section lamella from each sample was prepared in a FEI Helios NanoLab 450S FIB-SEM.

Experimental measurement of the thermal conductivity of the SLs by the 3ω method.

The cross-plane thermal conductivity of the SLs was measured by the 3ω method.[8,9] This is based on the use of a metallic resistor deposited on top of the sample to study, which acts, simultaneously, as a heater and a thermometer. An alternate current (of frequency ω) flows through the resistor, leading to a temperature oscillation in the film+substrate, due to Joule heating, which can be approximated, for a resistor of width w and with a penetration depth much smaller than the width of the resistor, by:

$$\Delta T \approx \frac{P}{\pi l \kappa}\left[\frac{1}{2}\ln\left(\frac{4D}{w^2}\right) - \frac{1}{2}\ln(2\omega) + \ln(2) + \eta - \frac{i\pi}{4}\right] \qquad \text{(S2)}$$

P is the power dissipated through the resistor, and η is the Euler-Mascheroni constant. With the change in temperature there will be associated a variation in the resistance of the metal heater at 2ω, as the resistance is not independent of temperature ($\mathrm{d}R/dT \neq 0$). Applying Ohm's law taking into account that the resistance has a component that is a function of 2ω and the current is a function of ω, the voltage will have a component at 3ω, which will be directly related to the heating of the resistor:

$$\boldsymbol{V = I_0\, sin(\omega t)\, R = I_0\, sin(\omega t)\left[R_0 + \frac{dR}{dT}\Delta T cos(2\omega t + \phi)\right] = I_0 R_0\, sin(\omega t) + I_0 \frac{dR}{dT}\frac{\Delta T}{2} sin(\omega t + \phi) - I_0 \frac{dR}{dT}\frac{\Delta T}{2} sin(3\omega t + \phi)} \qquad \text{(S3)}$$

Thus, the third harmonic of the voltage ($V_{3\omega}$) is a direct probe of the heating of the sample, and therefore of its thermal conductance:

$$V_{3\omega} = \frac{I_0}{2}\frac{dR}{dT}\Delta T \qquad \text{(S4)}$$

Taking this into account, from the slope (m) of $V_{3\omega}/V_{1\omega}$vs $\ln(2\omega)$ plot, we can obtain the thermal conductivity of the sample:

$$\kappa = \frac{I_0^2}{4\pi l m}\frac{dR}{dT} \qquad \text{(S5)}$$

A thin film between the resistor and the substrate can be modeled as a thermal resistance in series added to the system. Since the thin film's thickness, t, is much smaller than the thermal penetration depth and its thermal conductivity, κ_{film}, is smaller than that of the substrate, a frequency independent term adds to the heating:[10]

$$\Delta T_{film} = \frac{P\cdot t}{w\cdot l\cdot \kappa_{film}} \qquad \text{(S6)}$$

In Figure S4 we show an example of an actual measurement of a substrate and film + substrate.

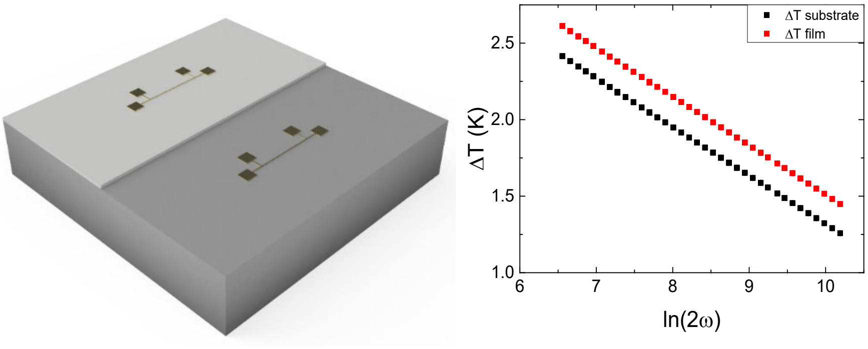

Figure S4. Left: Schematic of the resistances deposited on the film and substrate. Right: Measurement of the heating of a resistance deposited on top of an $SrTiO_3$ substrate (black) and another on top of a SL film, measured at 200 K. The thermal conductivity of the film is obtained from the offset between the two lines, according to equation S6.

For this work, the SLs were deposited by PLD covering only half the surface of the substrate; two resistors (10 nm Cr / 100 nm Pt; 1 mm long and 10 µm wide) were then deposited on the samples and on the film, so that the thermal conductivity of the two can be measured simultaneously.

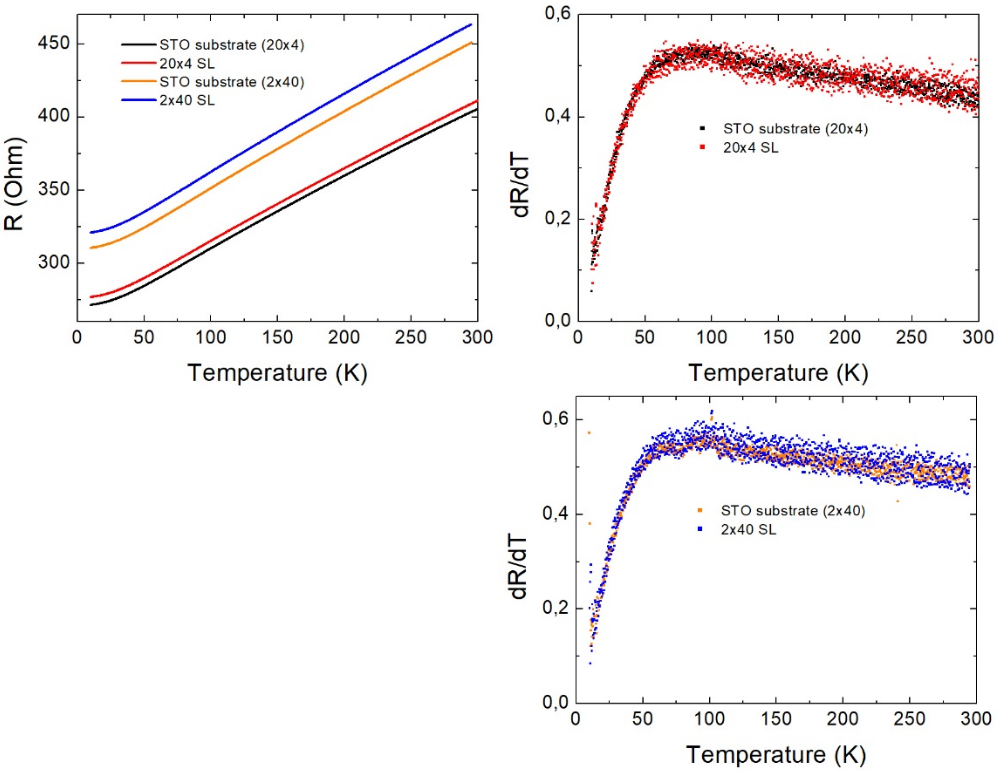

Figure S5. Temperature dependence of the electrical resistivity, and its derivative, for the resistances deposited on top of two SLs, and in the bare portion of the substrate.

LCO is not a perfect insulator; for that reason, as explained in the paper, our SLs always terminate in STO, which isolates the Pt sensor from the LCO.

Any leakage can be detected through a variation in the dR/dT of the Pt sensor. For that reason, we carefully checked this magnitude before every measurement of a SL. As you can see in figure S5, the R(T) and dR/dT of two Pt sensors on top of 2 SLs with different periodicity, as well as on top of a bare portion of the substrate, show the same behavior, within the error. This guarantees the absence of any measurable leakage through LCO, even with a 2 nm thick layer of STO (superlattice identified in the paper as SL 2x40).

To increase the accuracy, the first harmonic of the voltage was partially suppressed before entering the lock-in amplifier, using a specially designed circuit with three differential amplifiers and a set of resistors together with a variable resistor with PTC (positive temperature coefficient) close to zero.[11]

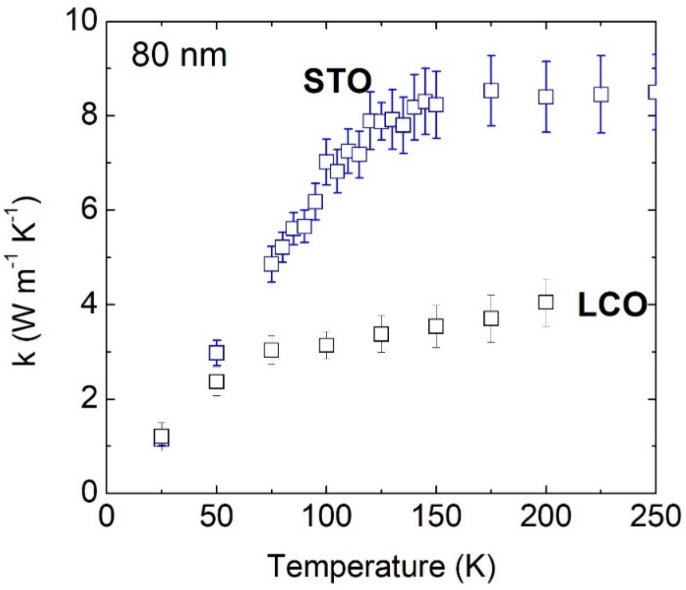

Figure S6. Temperature dependence of the thermal conductivity of 80 nm thick film of STO and LCO, respectively. To increase thermal contrast, the STO film is deposited on a MgO substrate.

We measured 80 nm thick films of pure LCO and pure STO (Figure S6). The 80 nm thick film of STO gives a value perfectly comparable with a film measured by Ravichandran et al.[12]; in this case synthesized by MBE and measured by time domain thermoreflectance. This supports the use of PLD and 3w for the synthesis and reliable measurement of the k(T) in oxide SLs.

References

(1) Fumega, A. O.; Fu, Y.; Pardo, V.; Singh, D. J. Understanding the Lattice Thermal Conductivity of SrTiO3 from an Ab Initio Perspective. *Phys. Rev. Mater.* **2020**, *4* (3), 033606. https://doi.org/10.1103/PhysRevMaterials.4.033606.

(2) Hohenberg, P.; Kohn, W. Inhomogeneous Electron Gas. *Phys. Rev.* **1964**, *136* (3B), B864. https://doi.org/10.1103/PhysRev.136.B864.

(3) Kohn, W.; Sham, L. J. Self-Consistent Equations Including Exchange and Correlation Effects. *Phys. Rev.* **1965**, *140* (4A), A1133. https://doi.org/10.1103/PhysRev.140.A1133.

(4) Perdew, J. P.; Burke, K.; Ernzerhof, M. Generalized Gradient Approximation Made Simple. *Phys. Rev. Lett.* **1996**, *77* (18), 3865. https://doi.org/10.1103/PhysRevLett.77.3865.

(5) Kresse, G.; Furthmüller, J. Efficient Iterative Schemes for *Ab Initio* Total-Energy Calculations Using a Plane-Wave Basis Set. *Phys. Rev. B* **1996**, *54* (16), 11169. https://doi.org/10.1103/PhysRevB.54.11169.

(6) Gonze, X.; Lee, C. Dynamical Matrices, Born Effective Charges, Dielectric Permittivity Tensors, and Interatomic Force Constants from Density-Functional Perturbation Theory. *Phys. Rev. B* **1997**, *55* (16), 10355. https://doi.org/10.1103/PhysRevB.55.10355.

(7) Li, W.; Carrete, J.; Katcho, N. A.; Mingo, N. ShengBTE: A Solver of the Boltzmann Transport Equation for Phonons. *Comput. Phys. Commun.* **2014**, *185* (6), 1747–1758. https://doi.org/10.1016/J.CPC.2014.02.015.

(8) Cahill, D. G.; Fischer, H. E.; Klitsner, T.; Swartz, E. T.; Pohl, R. O. Thermal Conductivity of Thin Films: Measurements and Understanding. *J. Vac. Sci. Technol. A Vacuum, Surfaces, Film.* **1989**, *7* (3), 1259–1266. https://doi.org/10.1116/1.576265.

(9) Cahill, D. G.; Pohl, R. O. Thermal Conductivity of Amorphous Solids above the Plateau. *Phys. Rev. B* **1987**, *35* (8), 4067–4073. https://doi.org/10.1103/PhysRevB.35.4067.

(10) Cahill, D. G.; Katiyar, M.; Abelson, J. R. Thermal Conductivity of A-Si:H Thin Films. *Phys. Rev. B* **1994**, *50* (9), 6077–6081. https://doi.org/10.1103/PhysRevB.50.6077.

(11) López-Bueno, C.; Bugallo, D.; Leborán, V.; Rivadulla, F. Sub-ML Measurements of the Thermal Conductivity and Heat Capacity of Liquids. *Phys. Chem. Chem. Phys.* **2018**, *20* (10), 7277–7281.

(12) Ravichandran, J.; Yadav, A. K.; Cheaito, R.; Rossen, P. B.; Soukiassian, A.; Suresha, S. J.; Duda, J. C.; Foley, B. M.; Lee, C.-H. H.; Zhu, Y.; Lichtenberger, A. W.; Moore, J. E.; Muller, D. A.; Schlom, D. G.; Hopkins, P. E.; Majumdar, A.; Ramesh, R.; Zurbuchen, M. A. Crossover from Incoherent to Coherent Phonon Scattering in Epitaxial Oxide Superlattices. *Nat. Mater.* **2014**, *13* (2), 168–172. https://doi.org/10.1038/nmat3826.
